# Intubation of the Larynx

**Published:** 1891-09

**Authors:** 


*Intubation of the Larynx.* By JAMES B. BALL, M.D. Pp. 54. London: H. K. LEWIS. 1891.

This little work forms a conveniently-arranged *resumé* of the literature bearing on the subject of intubation of the larynx. After briefly reviewing the history of the operation and the various devices by which O'Dwyer overcame the many practical difficulties he encountered, the author describes the instruments and the method of using them, together with the difficulties that may occur to the operator and how they may be met. The after-management of the patient, upon which success or failure very much depends, has a short chapter to itself.

We should have felt a greater interest in the work had there been any evidence that it was based on a personal experience of intubation and its difficulties; still, it will prove useful as an efficient guide for those who desire to become acquainted with the subject.

